# Bacterial Cellulose Membrane Containing *Epilobium angustifolium* L. Extract as a Promising Material for the Topical Delivery of Antioxidants to the Skin

**DOI:** 10.3390/ijms22126269

**Published:** 2021-06-10

**Authors:** Anna Nowak, Paula Ossowicz-Rupniewska, Rafał Rakoczy, Maciej Konopacki, Magdalena Perużyńska, Marek Droździk, Edyta Makuch, Wiktoria Duchnik, Łukasz Kucharski, Karolina Wenelska, Adam Klimowicz

**Affiliations:** 1 Department of Cosmetic and Pharmaceutical Chemistry, Pomeranian Medical University in Szczecin, Powstańców Wielkopolskich Ave. 72, 70-111 Szczecin, Poland; anowak@pum.edu.pl (A.N.); wiktoria.duchnik@pum.edu.pl (W.D.); lukasz.kucharski@pum.edu.pl (Ł.K.); adklim@pum.edu.pl (A.K.); 2 Department of Chemical Organic Technology and Polymeric Materials, Faculty of Chemical Technology and Engineering, West Pomeranian University of Technology in Szczecin, Piastów Ave. 42, 71-065 Szczecin, Poland; Edyta.Makuch@zut.edu.pl; 3 Department of Chemical and Process Engineering, Faculty of Chemical Technology and Engineering, West Pomeranian University of Technology in Szczecin, Piastów Ave. 42, 71-065 Szczecin, Poland; Rafal.Rakoczy@zut.edu.pl (R.R.); Maciej.Konopacki@zut.edu.pl (M.K.); 4 Department of Experimental and Clinical Pharmacology, Pomeranian Medical University in Szczecin, Powstańców Wielkopolskich Ave. 72, 70-111 Szczecin, Poland; magdalena.peruzynska@pum.edu.pl (M.P.); marek.drozdzik@pum.edu.pl (M.D.); 5 Department of Nanomaterials Physicochemistry, Faculty of Chemical Technology and Engineering, West Pomeranian University of Technology in Szczecin, Piastów Ave. 45, 70-311 Szczecin, Poland; Karolina.Wenelska@zut.edu.pl

**Keywords:** bacterial cellulose, plant extracts, antioxidant activity, phenolic acids, bioavailability, penetration skin

## Abstract

Bacterial cellulose membranes (BCs) are becoming useful as a drug delivery system to the skin. However, there are very few reports on their application of plant substances to the skin. *Komagataeibacter xylinus* was used for the production of bacterial cellulose (BC). The BC containing 5% and 10% ethanolic extract of Epilobium angustifolium (FEE) (BC-5%FEE and BC-10%FEE, respectively) were prepared. Their mechanical, structural, and antioxidant properties, as well as phenolic acid content, were evaluated. The bioavailability of BC-FESs using mouse L929 fibroblasts as model cells was tested. Moreover, In Vitro penetration through the pigskin of the selected phenolic acids contained in FEE and their accumulation in the skin after topical application of BC-FEEs was examined. The BC-FEEs were characterized by antioxidant activity. The BC-5% FEE showed relatively low toxicity to healthy mouse fibroblasts. Gallic acid (GA), chlorogenic acid (ChA), 3,4-dihydroxybenzoic acid (3,4-DHB), 4-hydroxybenzoic acid (4-HB), 3-hydroxybenzoic acid (3-HB), and caffeic acid (CA) found in FEE were also identified in the membranes. After topical application of the membranes to the pigskin penetration of some phenolic acid and other antioxidants through the skin as well as their accumulation in the skin was observed. The bacterial cellulose membrane loaded by plant extract may be an interesting solution for topical antioxidant delivery to the skin.

## 1. Introduction

The development of natural biomaterials for medical purposes has been observed in recent years. The application of natural, safe, and ecological vehicles for dermatological and cosmetic products could be important to reduce synthetic product use. The cellulose produced by bacteria, including *Komagataeibacter xylinus*, seems to be a promising material [1,2]. Bacterial cellulose membrane (BC) is characterized by high purity (lack of lignin, hemicellulose, and pectin), high degree of polymerization, high porosity, beneficial mechanical properties, high crystallinity, good moldability, biocompatibility, good permeability, resistance to degradation, high water absorption capacity (more than 90% of its weight) and are environmentally friendly [3,4,5,6,7,8]. These properties, together with tensile strength, make these membranes applicable as a skin repair material [9] and in wound dressing [10,11]. The BC is well tolerated by the skin and does not irritate [3]. In some studies, the BCs combination with other ingredients to enhance their therapeutic application were evaluated. Among the promising ingredients to be used to enrich the BC formulation are plant extracts. In recent years gradually increasing demand for to use of “natural” products, perceived by patients as safer compared to products containing “synthetic” ingredients has been observed [12]. Furthermore, “green” polymers, are an environmentally friendly alternative to synthetic materials to reduce a large amount of non-biodegradable waste generated by industry [6].

When the BCs are modified by incorporating other components as bioactive compounds, new activities such as antioxidant, antimicrobial, and anti-inflammatory can be observed. Moreover, these modifications can improve structural properties and biocompatibility [13]. In the literature, there are reports of combining BC with various plant extracts, including among others *Euclea schimperi* [14], *Boswellia serrata* [15], *Camellia sinensis*, *Hibiscus sabdariffa*, *H. rosa-sinensis* [16], pomegranate peel extract, green tea extract, rosemary extract [17], *Scrophularia striata* [18], *Zingiber officinale* root [19], and papain [20]. However, there are few reports on their application to deliver plant substances to the skin. To date, most studies have focused on the use of BC for transdermal delivery of drugs, such as diclofenac [21], lidocaine, ibuprofen [22], caffeine [23], silver sulfadiazine [24], and amoxicillin [25]. Only a few studies described the penetration of plant constituents from BC. Taokaew et al. evaluated the release of α-mangostin included in BC [26]. The issue of the permeation of active substances from BC is important because plant substances can accumulate in the skin, showing local effects, or penetrate through it into the underlying tissues.

Fireweed (*Epilobium angustifolium* (L.) Holub) (*Onagraceae*) is a well-known medicinal plant that grows naturally in many locations [27,28,29]. Recently, more and more attention has been paid to the use of this plant as a potential therapeutic agent in the treatment of various skin diseases and also as an active ingredient in cosmetics. It is due among other to its anti-inflammatory [30,31], antibacterial [30,32], anticancer, and analgesic [29,33], antioxidant properties [33,34,35]. Its pharmacological activity is related, among others, to the content of several bioactive compounds such as phenolic acids (PhA), including benzoic acid derivatives, e.g., GA, 3,4-DHB, 4-HB, 3-HB, and cinnamic acid derivatives, e.g., CA [30,36,37]. Phenolic acids and other antioxidants contained in the plants are valuable ingredients with antioxidant properties [38] in preparations applied to the skin and mucosa [37]. Karakaya reported a potent wound healing of *E. angustifolium* related to its antioxidant activity [35]. On the other hand, Zagórska-Dziok mentioned *E. angustifolium* as a potential plant to be used in anti-aging cosmetics. These authors demonstrated the cytoprotective properties of E. angustifolium extracts on skin cells, keratinocytes, and fibroblasts [31]. Taking into account all the above-mentioned properties, this plant seems to be a good candidate for inclusion in BC to create a natural film to be used in dermatology and cosmetology.

This study aimed to investigate the potential of BC-FEEs as a system for topical delivery of valuable antioxidant substances to the skin.

## 2. Results

### 2.1. Chemical Composition and Antioxidant Activity of the FEEs

**Figure 1:**
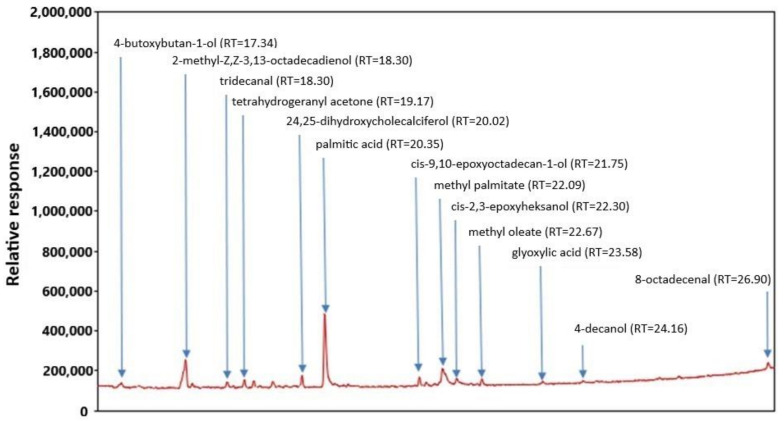
GC-MS chromatogram of FEE.

The gas chromatography-mass spectrometry (GC-MS) chromatogram of FEE is presented in Figure 1. The analysis of extracts showed the content of 13 main compounds: tetrahydrogeranyl acetone, palmitic acid, cis-9,10-epoxyoctadecan-1-ol, methyl palmitate, cis-2,3-epoxyheksanol, glyoxylic acid, and 8-octadecenal, 4-butoxybutan-1-ol, 2-methyl-Z,Z-3,13-octadecadienol, tridecanal, 24,25-dihydroxycholecal, methyl oleate, and 4-decanol (Figure 1). 

**Figure 2:**
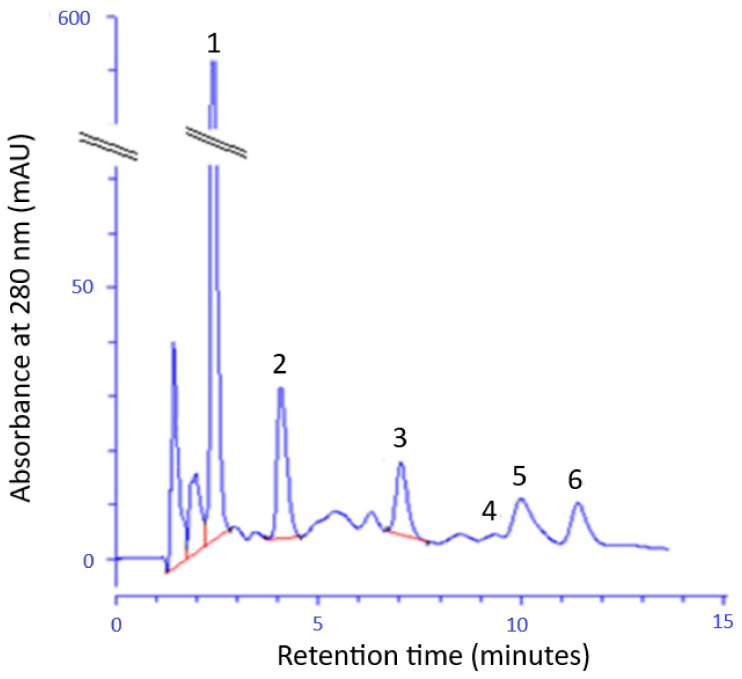
The example chromatogram of phenolic acids identified in the FEE; gallic acid (1), 3,4-dihydroxybenzoic acid (2), 4- hydroxybenzoic acid (3), 3- hydroxybenzoic acid (4), chlorogenic acid (5), and caffeic acid (6).

The HPLC method was used to identify and quantification of selected phenolic acids in FEE (Figure 2). The following phenolic acids were found: GA; ChA; 4-HB; 3-HB and 3,4-DHB and CA. The GA, 4-BH, and ChA were identified in the largest amount.

The tested plant extracts were characterized by a high total polyphenol content as well as antioxidant activity, measured by the Folin-Ciocalteu, DPPH, and ABTS methods. It was observed that these parameters increase with the increase of the plant concentration in the extract (Table 1).

### 2.2. The TG, DTG, FTIR SEM, and Mechanical Properties of BC and BC-FEEs

**Figure 3:**
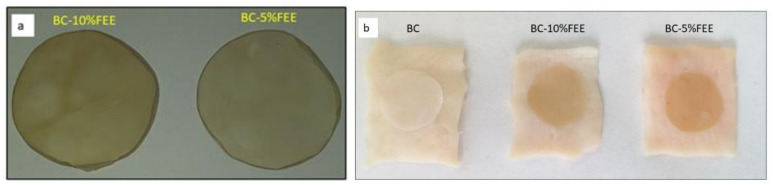
The BC-10%FEE and BC-5%FEE dry membranes (**a**), the all membranes before mounting in the Franz diffusion cell (**b**).

The obtained membranes were homogeneous as shown in Figure 3a and also showed good adhesion to the skin (Figure 3b).

**Figure 4:**
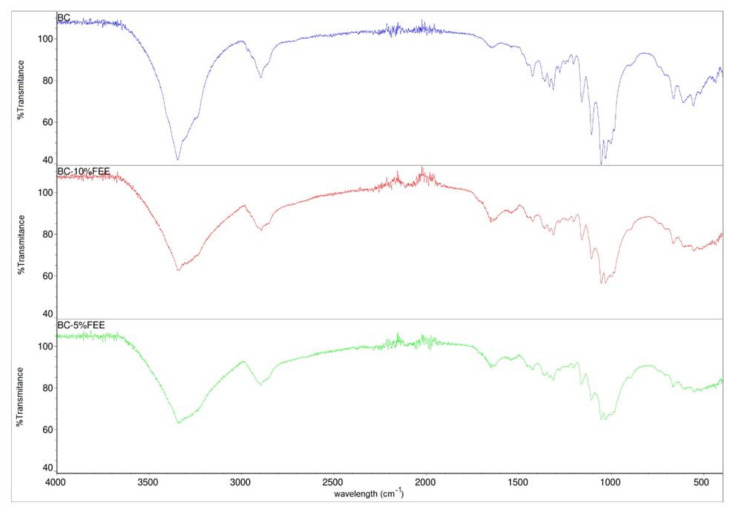
FTIR-ATR spectra of bacterial cellulose—BC (violet), BC-10%FEE (red) and BC-5%FEE (green).

The FTIR spectra of BC, BC-5%FEE, and BC-10%FEE are presented in Figure 4. The FTIR spectrum of bacterial cellulose shows typical absorption bands characteristic for cellulosic materials. The groups O-H, C-H, and C-O-C are visible at 3346, 2895, and 1161 cm^−1^, respectively. Furthermore, the weak and broadband centered at approximately 897 cm^−1^ and strong band centered at approximately 1426 cm^−1^ which can be assigned to CH_2_ bending vibration, defined the cellulose as cellulose I, which suggested that BC produced in this study could be pure cellulose. The spectra of BC-FEEs are very similar to the pure BC spectrum. The increase in the intensity of the bands at range 1500–1750 cm^−1^ in cases of BC-FEEs was observed (Figure 4). The FTIR spectra collected at different points of the surface and inner layers of FEE-loaded membranes showed a similar profile.

**Figure 5:**
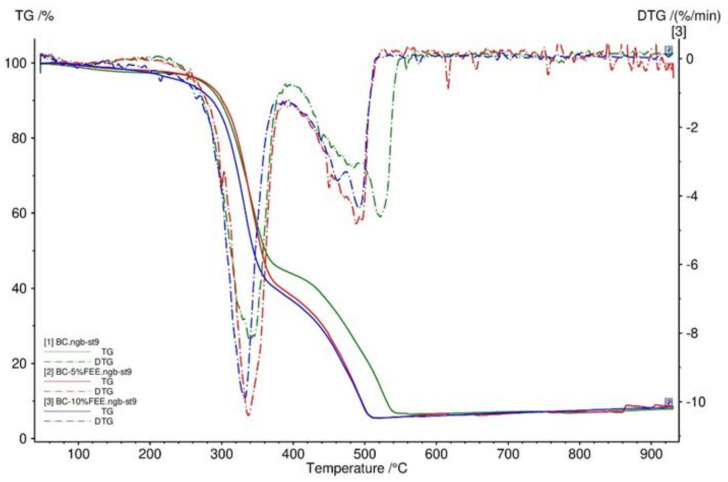
TG and DTG curves of BC membranes—BC (green), BC-10%FEE (blue), and BC-5%FEE (red).

To determine the thermal decomposition behavior of BC membranes, TG was performed. Figure 5 showed that the TG and DTG curves had very similar trends for BC and BC-5%FEE and BC-10%FEE. Three distinct steps were recorded for the weight loss of the BC membranes, namely the weight loss of around 100 °C, 340 °C, and 450 °C. All obtained membranes were very stable and had no degradation up to 200 °C as well as had a comparable residual mass (Figure 5). 

**Figure 6:**
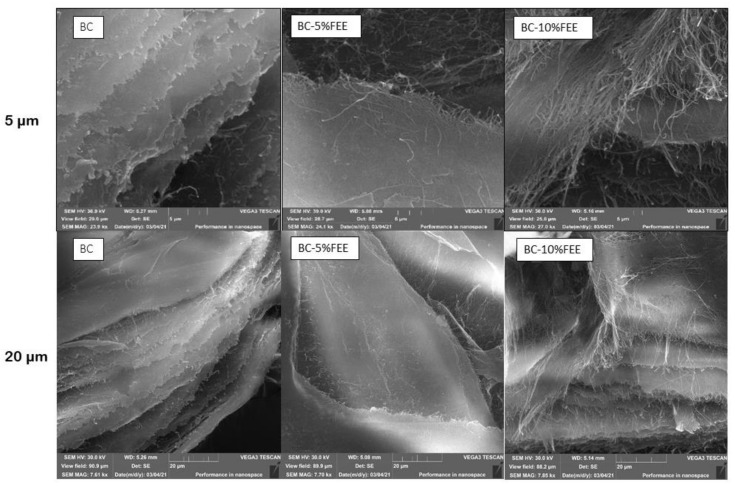
The SEM micrographs of BC, BC-5%FEE, and BC-10%FEE on a scale of 5 and 20 µm.

The SEM images of the dried BC, BC-5%FEE, and BC-10%FEE are shown in Figure 6. From the SEM image, the surface of BC was composed of many neat fibrils and makes an aggregated structure. In all membranes, the characteristic tridimensional network was observed. The nanofibrils of the BC-5%FEET and BC-10%FEE are longer compared to pure BC, while in the case of BC-10%FEE there are more of them (Figure 6).

Table 2 summarizes the mechanical properties of the obtained membranes such as Young’s modulus, tensile strength, and elongation at break. The mechanical properties of BC with and without FEEs are comparable. The best properties were shown by BC-5%FEE (Table 2).

### 2.3. Antioxidant Properties of Phenolic Acids Contain in BC and BC-FEEs

Table 3 presents the concentration of phenolic acids in BC-FEEs as well as BC-FEEs antioxidant activity and the total polyphenol content. The analyzed membranes contained selected phenolic acids and were also characterized by antioxidant activity, measured by DPPH and ABTS methods. The following phenolic acids have been identified: ChA, GA, 4-HB, 3-BH, 3,4-DHB, and CA. GA was identified in the highest amount—453.66 ± 6.95 µg/g membrane for BC-10% FEE and 275.44 ± 56.44 µg/g membrane for BC-5% FEE while CA was only 57.80 ± 4.12, and 31.83 ± 1.23 µg/g membrane, respectively. The phenolic acids were not found in pure BC (Table 3). Both membranes were characterized by antioxidant activity, evaluated with the DPPH method—0.55 ± 0.01 mmol Trolox/l and 2.09 ± 0.01 mmol Trolox/l for ABTS, while the total polyphenol content determined by the Folin-Ciocalteu method was 0.63 ± 0.02 mmol GA/l for BC-10%FEE. Lower activity of 0.44 ± 0.01 mmol Trolox/l for DPPH and 1.59 ± 0.01 mmol Trolox/l for ABTS and 0.45 ± 0.01 mmol GA/l determined by the Folin-Ciocalteu method was found for BC-5%FEE. The pure BC did not show any antioxidant activity nor total polyphenol content (Table 3).

### 2.4. Biocompatibility Study

**Figure 7:**
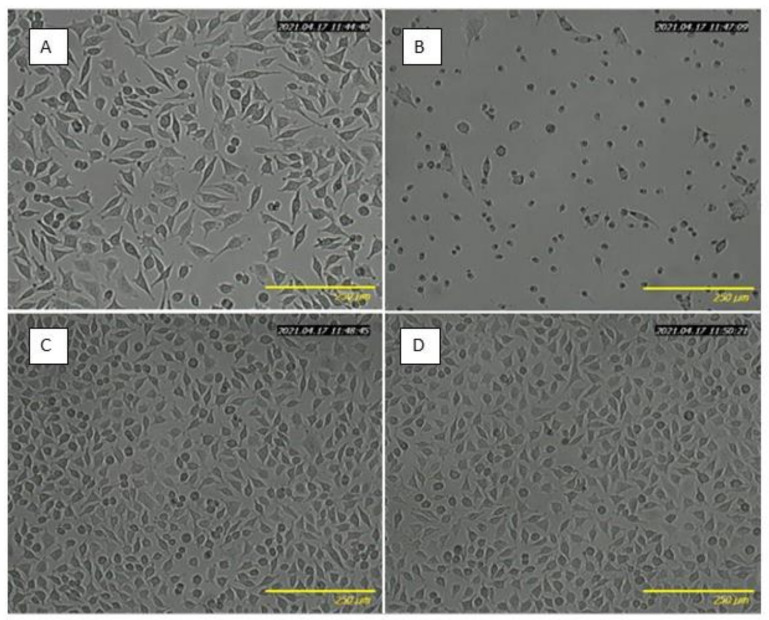
Optical microscopy images of L929 cells after 24 h incubation with medium containing extracts from BC-5%FEE (**A**), BC-10%FEE (**B**), BC (**C**), and control (**D**).

Table 4 shows the total phenolic content released from BC-5% FEE, BC-10% FEE, and BC to the culture medium and its effect on cell viability. After 24 h of incubation, the WST-1 assay results demonstrated the biocompatibility of pure BC (with cell viability equal to 100%), and dose-dependent cytotoxicity of the released FEEs extracts. The release rate was analyzed in terms of the total phenolic content as the main bioactive compounds. The extract released from BC-5%FEE, containing 0.3 mmol GA/l, decreased cell viability to 73.99 ± 6.42%. Whereas, extract from BC-10%FEE, containing two times higher amounts of polyphenols, significantly reduced cell viability to 14.16 ± 10.07%. The obtained results were consistent with microscopy imaging, where lower cell density under 5%FEE exposure, higher cytotoxicity in 10%FEE treated cells, and no differences between pure BC-treated and the control cells, were observed (Figure 7).

### 2.5. In Vitro Penetration Studies

Table 5 summarizes the content of selected phenolic acids in the acceptor fluid collected after 24 h penetration as well as the antioxidant activity of the studied fluid. 

**Figure 8:**
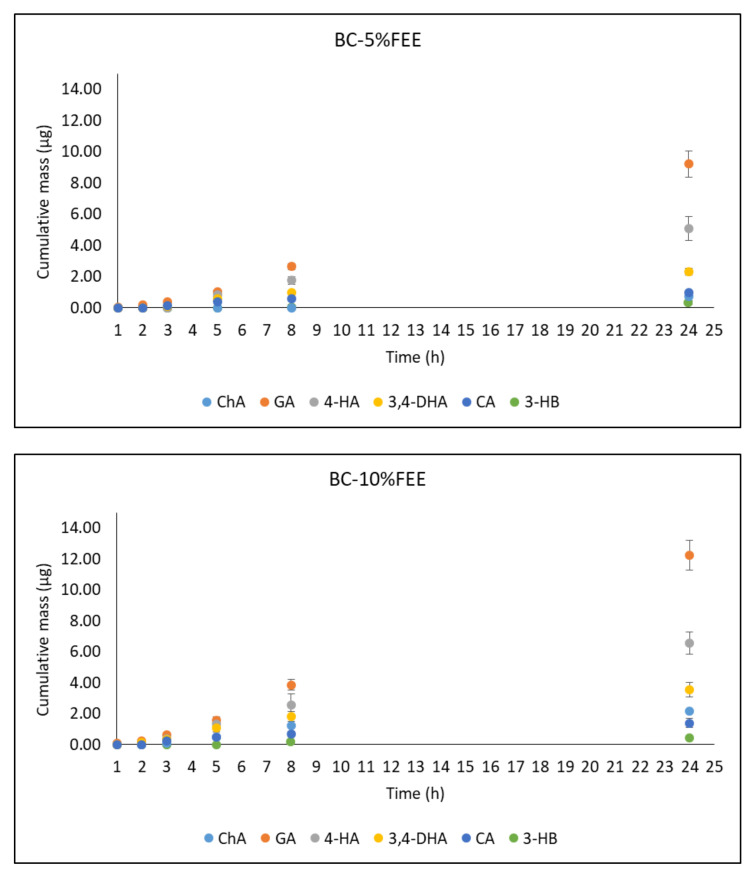
The cumulative mass of phenolic acids in the acceptor fluid during the 24 h penetration. Vertical lines present standard deviation. *n* = 6.

Figure 8 shows the cumulative mass of phenolic acids in the acceptor fluid collected during the entire experiment. In the case of the BC-10%FEE, GA, and 4-HB penetrated to a higher degree than other acids. The cumulative amounts of these acids after the 24 h study were 12.26 ± 1.96 and 6.56 ± 0.09 µg, respectively. A similar tendency was observed for penetration of BC-5%FEE, the respective amounts were: 9.22 ± 0.84 and 5.08 ± 0.78 µg. The acceptor fluid, collected after the end of penetration showed antioxidant activity only with the ABTS and Folin-Ciocalteu methods. (Table 5).

The summary of the permeability parameters for BC-FEEs is shown in Table 6. The highest permeation rate of 0.55 ± 0.08 μg cm^−2^ h^−1^ were observed for GA from BC-10%FEE. However, the best penetration characteristic as a whole was found for 3,4-DHB. On the contrary, the lowest permeation parameters including the lowest K_p_ and the lowest J_ss_ was found for 3-HB, which was only detectable after 5 h or 24 h of permeation study in BC-10%FEE and BC-5%FEE, respectively. The lowest lag time was noticed for 3,4-DHB and the highest for 3-HB (Table 6).

The transdermal diffusion study showed that all of the phenolic acids evaluated were cumulated into the pigskin. The samples obtained after skin extraction following 24 h penetration were characterized by the antioxidant activity of 0.39 ± 0.01 and 1.52 ± 0.06 mmol Trolox/l for DPPH and ABTS methods, respectively, for BC-10%FEE and 0.27 ± 0.01 and 1.02 ± 0.01 mmol Trolox/l evaluated with DPPH and ABTS methods, for BC-5%FEE. A similar tendency for the total polyphenol content was observed. The GA and 4-HB cumulated to a higher degree than the others, 222.94 ± 16.60 µg/g skin and 127.67 ± 1.59 µg/g skin, respectively, for BC-10%-FEE as well as 151.34 ± 13.85 µg/g skin and 45.36 ± 1.33 µg/g skin for BC-5%FEE—Table 7.

## 3. Discussion

In recent years, the use of cellulose membranes to deliver therapeutic agents to the skin has gradually attracted more attention, due to their ease and safety application, as well as their biodegradability [3,39] and biocompatibility [39,40]. Increasingly, attempts have been made to incorporate into BC natural plant extracts containing components with antioxidant properties. *E. angustifolium* has been used for a long time in folk medicine as an herb supporting the treatment of skin infections [35]. This plant is characterized by high antioxidant activity and contains many valuable compounds, including phenolic acids [31,41]. It is worth mentioning that these compounds penetrated to human skin from the ethanolic extract of this plant, as we demonstrated in our previous study [42]. Therefore, due to its high antioxidant activity and valuable components, we have selected *E. angustifolium* as a raw material for inclusion in BC.

In the first stage, before including FEEs into BC, we estimated their chemical composition and antioxidant activity. The analysis by GC-MS showed the content of several groups of compounds, as tetrahydrogeranyl acetone, palmitic acid, cis-9,10-epoxyoctadecan-1-ol, methyl palmitate, cis-2,3-epoxyheksanol, glyoxylic acid, and 8-octadecenal. Fatty acids were the relevant components of the extract. The presence of methyl esters of fatty acids (methyl palmitate and methyl oleate) was also confirmed by others in the extracts of dried and fresh leaves of *E. angustifolium* [43] and essential oils from *E. angustifolium* [44] and *E. hirsutum* [45]. Among seventeen major components were identified by GC-MS in ethanol extracts of *E. montanum* by Canli et al., fatty acids were a large group [46]. Other organic compounds of the aldehydes group, among other 2-decanal, hexanal, octanal, N-nonanal, as well as alcohols have been identified by Jariene et al. [47]. The content of volatile compounds in the plant is primarily affected by the vegetation phase of plants, meteorological conditions, plant chemotype, and methods of obtaining the extract including the solvent used in extraction [33,42,43]. In our study the following phenolic acids were found in FEE: GA; ChA; 4-HB; 3-HB, 3,4-DHB, and CA, of which the highest concentrations of GA, 4-BH, and ChA were observed. Some phenolic acids, such as GA, ChA CA were also identified in leaves of *E. angustifolium* by Agatonovic-Kustin et al., Ferrante et al., Ruszová et al., and Lasinskas et al. [30,33,48,49]. Shikov et al. and Zagórska-Dziok et al. found a high amount of GA in *E. angustifolium* extract [31,50], this result was confirmed in our study. This phenolic acid was also found in other varieties of *Epilobium*, such as *E. hirsutum* and *E. partiflorum* [51]. The phenolic acids contained in *E. angustifolium*, belong to the group of hydroxycinnamic acids (ChA and CA) as well as to the group of hydroxybenzoic acids (GA, 4-BH, 3-HB, and 3,4-DBH), and are characterized by anti-inflammatory and antibacterial properties [38]. These compounds are also responsible for strong antioxidant capacity. The results of many studies confirmed the positive correlation between the antioxidant activity and the content of phenolic acids or other polyphenols [42,52]. Therefore, in our study, we decided to evaluate the antioxidant activity of the analyzed extracts incorporated into BC. The FEEs were characterized by high antioxidant potential and total polyphenols content, as confirmed by others [31,33,34,53,54,55]. Similar results were also observed for other *Epilobium* varieties, such as *E. roseum*, *E. parviflorum*, *E. hirsutum*, *E. adenocaulon*, *E. montanum, E. palustre* [34,42,55,56]. The antioxidant activity is an important factor in skincare and regeneration, due to tissue damage and cell degradation by free radicals (RFTs) [31,57,58]. Moreover, the scavenging of free radicals could prevent bacterial skin infections, since bacterial infections depend also on oxidative stress [59]. The oxidative stress can increase the infection severity and could disturb wound healing [60]. Due to the high antioxidant activity and the content of valuable compounds, *E. angustifolium* extracts can be included in dermatological and cosmetic preparations, having positive effects on the skin [31,61]. Antioxidant capacity of, for example, ointment contained leave extract is used auxiliary to treat skin diseases in children, moreover, root compresses could be helpful in the treatment of burns, swelling, boils, and skin irritations [35]. *E. angustifolium* extracts action based on the inhibition of the activity of lipoxygenase, elastase, and collagenase as well as a protective effect on keratinocytes and fibroblasts [31,49,61].

In our study, a simple technique to enrich BC with FEEs at two concentrations—5% and 10%, based on soaking for 24 h at room temperature was applied. This method of processing was also used by others. Taokaew et al. immersed the purified BC films into the ethanolic mangosteen extract solutions under ambient conditions for 24 h [26]. Swinger et al. confirmed, that the most common method of loading drugs in BC membranes is their immersion in the drug solution [9]. This is a safe and low-cost way to incorporate the drug or plant ethanol extract into the BC film [19]. The BC containing FEEs prepared in our study were homogeneous, indicating that FEE was well dispersed inside the tridimensional BC nanofibers, and also showed good adhesion to the skin. Moreover, the FTIR spectrum confirmed the purity of the BC as well as of the included plant extracts. The FTIR analysis of bacterial cellulose shows typical absorption bands characteristic for cellulosic materials. After the incorporation of FEEs into the BC, an increase in the intensity of the bands at range 1500–1750 cm^−1^ was observed, which can be attributed to the C=O stretching vibration or the C=C stretching vibration probably from the compounds presented in FEEs, in particular tetrahydrogeranyl acetone, methyl palmitate, methyl oleate, glyoxylic acid, and 8-octadecenal. Moreover, FTIR spectra collected at different points of the surface and inner layers of FEEs-loaded membranes showed a similar profile, confirming the good dispersion of extract ingredients inside the membranes.

In our study, three distinct steps were recorded for the weight loss of the BC membranes.

The weight loss of around 100 °C was due to the evaporation of adsorbed water, which indicated that pure BC had more adsorbed water than BC-5%FEE and BC-10%FEE. This effect is due to the drying effect of ethanol. It is related to the formation of a water/ethanol azeotrope which can be removed at a lower temperature than water alone. The major peaks, observed around 340 °C, were caused by cellulose degradation processes, such as depolymerization, dehydration, and decomposition of glucosyl units followed by the formation of charred residues. Other authors observed maximum rates of weight loss for bacterial cellulose to occur at 360–390 °C [62,63]. The third weight loss above 450 °C showed the oxidation and breakdown of the charred residues to lower molecular weight gaseous products [64]. The differences in thermogravimetric results may be due to several factors, as sample preparation, sample size, morphology, and homogeneity [65]. Furthermore, all obtained membranes were very stable and had no degradation up to 200 °C. It has been noticed that the thermal stability of bacterial cellulose with *E. angustifolium* extract was lower than that without the extract. This is probably due to the presence of compounds such as methyl esters of fatty acids (methyl palmitate and methyl oleate) in the extract used. All membranes obtained had a comparable residual mass.

We observed from the SEM image, that the surface of BC was composed of many fibrils and formed an aggregated structure. The nanofibrillars of the BC with plant extracts are longer and denser, especially in BC-10% FEE. This is probably due to the high concentration of the plant in the extract and the deposition of the active substances inside the membrane. Taokew et al. reported an increase in BC thickness while loading them with a mango extract. They suggested that plant particles penetrated deeply and filled the pores of BC [26]. Similar changes in morphology were found in the BC-containing papain solution. The BC fibrils had changed from having a smooth surface to being rough with many small particles attached to the cellulose fibrils [20].

The good mechanical properties of BC make it an attractive material for tissue regeneration [4]. In our study, the mechanical properties of BC with and without FEEs were comparable, however, lower strength was observed for BC-10% FEE. Similarly, increasing the mango extract in BC membranes resulted in a significant reduction of tensile strength of the dried membranes. However, for this study, these values were significantly higher after BC rehydration [26]. According to Ul-Islam et al., BC containing *Aloe vera* gel possessed 3-fold better mechanical strength than pure BC [11]. Likewise, the incorporation of the *Scrophularia striata* extracts caused significantly lower tensile strength and Young’s modulus compared to the control film (pure BC). The substances of plants in the BC membrane probably play a role as the plasticizer and reduced the interactions among the macromolecules, which in turn resulted in the decrease of the strength [18].

Incorporating plant extracts into BC may increase their antioxidant activity. In our study, it was observed antioxidant activity of pure membrane after adding FEE in two concentrations—5% and 10%. Antioxidant activity, total polyphenols content, as well as selected phenolic acids contained, were found in both membranes, however, these parameters were higher for BC-10% FEE as compared to BC-5% FEE. Moradian et al. obtained similar results on the antioxidant activity of bacterial cellulose membrane with rosemary extracts at two concentrations (25 and 50%) and similarly, the antioxidant activity significantly increased with increasing concentration of the extracts [17]. Fernandes et al. reported a high concentration of phenolic compounds and antioxidant activity of cellulose membranes containing grape pomace [13]. Taokaew et al. determined the total polyphenol content of BC films containing ethanolic mangosteen peel extract ranged from 2.06 to 248.20 mg GA/l of dried film [26]. BC containing 5% extract of *Scrophularia striata* also had high antioxidant activity [18]. The antioxidant activity of BC containing plant extracts may be due among others to the presence of phenolic compounds [18], with a high ability to scavenge free radicals [66,67]. Similarly, the addition of collagen into BC was also effective to scavenge ROS, showing nearly 80% antioxidant activity against the peroxynitrite anion (ONOO^−^) and superoxide anion (O_2_^−^) [68]. The antioxidant activity of BC may play an important role in the healing of chronic and burn wounds. Exudation from non-healing wounds is characterized by elevated levels of, among others, reactive oxygen species (ROS), leading to a reduced concentration of growth factors and proteinase inhibitors and degradation of tissues [68]. In our study, no antioxidant activity nor total polyphenol content was found in pure BC. High-purity BC does not exhibit antioxidant properties because pure cellulose with a high degree of crystallinity cannot inhibit free radicals [17]. Therefore, topical application of BC enriched with antioxidant components contained in plants seems to be an interesting option. The incorporation of natural active ingredients into BC can be used for topical regeneration and skincare [13,16]. The penetration of antioxidant substances deep into the skin or their accumulation in the skin also plays an important role. Evaluation of skin permeation of bioactive compounds is an important factor in the development of membranes for topical delivery of therapeutic agents [69]. The antioxidant activity of both the ethanol extract prior to loading into BC, as well as the membranes after plant extract loading was also evaluated. In both cases, the antioxidant activity was high during the study, which could confirm the high stability of the prepared cellulose membranes. Membranes loading with *E. angustifolium* extract contained individual phenolic acids, also identified after longer storage. This observation confirmed plant extracts’ stability.

Biocompatibility is defined as a capability of a compound or material to be therapeutically active once it is applied to a recipient without causing a systemic or local adverse response [9]. In our study, the cytotoxicity of BC-FEEs was evaluated In Vitro using the L929 murine fibroblasts. Our data confirmed the biocompatibility of pure BC and dose-dependent cytotoxicity of BC-FEEs related to the efficient release of the plant extracts in cell culture conditions. It should be emphasized that the amount of 0.3 mmol GA/l released from BC-5%FEE to the culture medium was equal to the amount of total polyphenol content accumulated in the skin during penetration study, and produced a moderate effect on fibroblast viability. As expected, BC-10%FEE showed the highest cytotoxicity resulting from a high dose of the plant extract loaded into BC. These findings are in keeping with several previous studies, where plant extracts used in too high doses produced a toxic effect on fibroblasts, significantly affecting their viability [70,71]. In future research, it seems to be necessary to select the optimal, cell non-toxic concentrations, while effectively accumulating in the skin.

In our study, an In Vitro skin penetration using porcine abdominal skin was conducted to determine the feasibility of using BC with FEE for topical and transdermal drug delivery. As human skin is not easily available it is often replaced by other skin and as a rule, porcine skin is frequently used for preliminary assessment of transdermal penetration of topically applied drugs. Numerous histopathological studies confirmed its similarity to human skin [72]. The antioxidant activity and total polyphenol content were evaluated in the analyzed samples obtained during the penetration test. Determinations were performed in acceptor fluid collected during 24 h penetration and in fluid obtained after skin extraction. The acceptor fluid showed antioxidant activity only with the ABTS and Folin-Ciocaleu methods. The reason for this was probably the low penetration of active substances through the skin, observed in the case of selected phenolic acids, with their greater accumulation in the skin. Similarly, Taokaew et al. demonstrated skin permeation of phenolic compounds and α-mangostin from BC films containing ethanolic mangosteen peel extract through pigskin. They found low permeation of the studied compounds into phosphate and acetate buffers, ranged from 0.1 to 1.6% [26]. However, they observed, that 95.6–99.5% of the phenolic compounds released from the films contained mango peel extract, were absorbed into the pigskin after 48 h penetration [26]. Phenolic acid permeation profiles are very useful to obtain the permeation parameters such as the steady-state permeation flux (J_SS_), the diffusion coefficient (K_P_), and the time required to reach steady-state permeation (lag time—L_T_). Taking into account these values, in our study, the most interesting compound is generally 3,4-DHB. However, most of the analyzed phenolic acids accumulate in the skin. For some drugs, faster and more efficient penetration is preferable, to achieve a rapid therapeutic effect. This mainly applies to anti-inflammatory and analgesic drugs [73]. On the other hand, in the case of some plant substances, their greater accumulation in the skin is preferred, as, through their antioxidant activity, they could show among others, the anti-aging effect [74]. It is obvious, that apart from the polyphenols, the skin may penetrate the whole pool of antioxidants contained in plants, however, phenolic acids constitute a significant part of skin-permeable compounds [42,52].

## 4. Materials and Methods

2,2-diphenyl-1-picrylhydrazyl (DPPH), 6-hydroxy-2,5,7,8-tetramethylchroman-2-carboxylic acid (Trolox), 2,2’-azino-bis(3-ethylbenzothiazoline-6-sulfonic acid) (ABTS), 2,4,6-tripyridyl-s-triazine (TPTZ), caffeic acid, chlorogenic acid, 3,4-dihydroxybenzoic acid, and WST-1 reagent were purchased from Sigma Aldrich (Poznań, Poland); Folin-Ciocalteu reagent, gallic acid, 4-hydroxybenzoic acid, 3-hydroxybenzoic acid, disodium phosphate and potassium dihydrogen phosphate from Merck, Darmstadt (Germany); sodium acetate anhydrous, potassium persulfate, potassium acetate, 99.5% acetic acid, aluminum chloride, 36% hydrochloric acid, sodium chloride, potassium chloride, citric acid, pH = 4 buffer, sodium hydroxide of high purity, ethanol and methanol were from Chembur (Piekary Śląskie, Poland), whereas acetonitrile for HPLC from J.T. Baker, (VWR Chemicals, Radnor, PA, USA). Yeast extract and Bacto Peptone were obtained from Graso BIOTECH (Starogard Gdański, Poland). D-mannitol (≥99.9%) was purchased in POL-AURA (Dywity, Poland). All reagents were of analytical grade.

Plant material was purchased from a local certified herbal store (HerbaPeru, Wrocław, Poland). The plant material was stored in a dark room and was dried at room temperature to a constant weight [54]. Samples were deposited in the plant material storage room (No. FEE-AM2020–10) at the Department of Cosmetic and Pharmaceutical Chemistry of the Pomeranian Medical University. Before extraction, the raw material was ground in the grinder, sieved using a circular-hole screen (8 mm mesh). 10 g and 5 g of dried raw material were extracted with 100 mL 70% (v/v) ethanol [75] for 60 min in an ultrasonic bath at a frequency of 40 kHz. Then, extracts were filtered through a Whatman paper filter (codified FEE03) and stored at +4 °C until analysis and then placed in BC.

Komagataeibacter xylinus bacteria strain from American Type Culture Collection (ATCC^®^ 53524™) was selected for bacterial cellulose production. For the bacteria cultivation, a modified buffered Hestrin-Schramm medium was used (Bacto Peptone 5.00 g/L, yeast extract 5.00 g/L, disodium hydrogen phosphate 2.70 g/L, citric acid monohydrate 1.15 g/L). Medium ingredients were soluted in distilled water and next autoclaved at 121 °C for 15 min. Then, the 20% *w/w* of the filter-sterilized aqueous solution of mannitol was added into the culture medium to reach a 20 g/L concentration of carbon source. Additionally, the medium was buffered to reach a pH level of 5.0. BC production was performed in rectangular plastic litter boxes (inner dimensions: 253 × 325 × 57 mm). Each box was filled with 1.2 l of medium and 100 µL inoculum, created from the original stock sample was added. Next, the litter box was wrapped with the food foil and moved into the incubator (30 °C) for 8 days. After this time the BC was harvested. Following this, BC was processed by washing in distilled water to remove the remaining content of the bacteria medium and then immersed in a hot (80 °C) 0.1 M aqueous solution of sodium hydroxide for 30 min to remove any bacteria cells that could stay on BC. After this procedure, BC was second time washed in distilled water until the pH = 7 was reached (which means all NaOH leftovers were removed).

Bacterial cellulose (BC) membranes were prepared by cutting round pieces of dimensions 140 × 8 mm, then were weighted and handily compressed to remove 50–60% of their water content. Drained BC membranes were then soaked in 5% and 10% (*v/v*) FEE extracts, for 24 h at room temperature to assure complete absorption of the extract. After the total absorption of the extract, the BC membranes were dried at 40 °C in a ventilated oven for 12 h. Next, all membranes were weighed, to determine the residue of the pure plant extract. BC membranes were prepared according to this method without adding FEE. The membranes were stored in a desiccator until use. The content of FEE in dried membranes is presented in Table 8. The content of the phenolic acids in the dried film samples was determined by HPLC.

The mechanical properties of BC samples were analyzed using Instron 5982 testing machine (Norwood, MA, USA) in tensile mode with a 1 kN load cell. The samples were cut into cuboid shapes. The dimensions of samples measured were length: 70 mm width: 10 mm and thickness: 0.3 mm. At least 5 specimens were tested from each sample. The corresponding stress (MPa) strain (%) curves were plotted, and Young’s modulus was determined.

SEM micrographs of all obtained BC membrane surfaces were obtained on operating at 15 kV. Samples were placed in steel support and coated with evaporated carbon. 2 samples of each membrane were analyzed.

Thermogravimetric analysis (TG and DTG) was carried out with TG 209 F1 Libra (Netzsch, Germany). All analyses were performed with a 5 mg sample in 6.8 mm (85 µL) crucibles under an air atmosphere between 25 and 1000 °C.

Fourier transform infrared (FTIR) spectra were obtained in a Thermo Scientific Nicolet 380 spectrometer (Waltham, MA, USA) equipped with an ATR diamond plate. Thirty-two scans were acquired in the 4000–400 cm^−1^ range with are a resolution of 4 cm^−1^.

Qualitative chemical analyses were performed using a GC–MS system comprised of TRACE GC series apparatus with a VOYAGER mass detector using a DB5 capillary column (30 m × 0.25 mm × 0.5 µm film thickness). The carrier gas was helium at a constant flow of 1.0 mL/min, sample chamber temperature of 240 °C, and a detector voltage of 350 V. The sample partition coefficient in the dispenser was 20, the volume of dispensed sample was 0.1 µL, and the ion mass range was 25–350 mV/z. The oven was held at 50 °C (2.0 min), then increased by 10 °C/min to 310 °C, and then cooled to 50 °C.

Phenolic acid concentrations in FEEs and BC-FEEs were determined by HPLC. To obtain the BC-FEEs samples for analysis the modified method by Moradian et al. was used [17]. Approximately, 100 mg of each membrane was immersed in 2 mL of methanol and incubated for 24 h at 4 ºC. After this time, the supernatant was collected and submitted for analysis. A similar procedure was also performed for pure BC [17]. The concentration was determined by high-performance liquid chromatography with ultraviolet detection (HPLC-UV) using an HPLC system from Knauer (Berlin, Germany). The test components were separated on a 125 mm × 4 mm column containing Hyperisil ODS (Thermo Scientific, Waltham, MA, USA), particle size 5 µm. The mobile phase consisted of 1% acetic acid, acetonitrile, methanol (50:40:10 by volume), the flow rate was 1 mL/min. 20 µL of the sample was injected onto the column. The column temperature was set at 25 °C. The correlation coefficient of the calibration curve was 0.996 (y = 277,926 x + 0.226, t_R_ = 2.402 min) for gallic acid; 0.999 for chlorogenic acid (y = 53,905 x + 9.831, t_R_ = 10.042 min); 0.999 for 4-hydroxybenzoic acid (y = 26,889 x + 3.5605, t_R_ = 6.960 min); 0.999 for 3,4-dihydroxybenzoic acid (y = 78,007 x − 1.1925, t_R_ = 4.194 min); 0.998 for 3-hydroxybenzoic acid (y = 15,214 x + 0.5775, t_R_ = 9.160 min); and 0.999 for caffeic acid (y = 67,950 x + 5.141, t_R_ = 11.275 min). All samples were analyzed in triplicate.

The scavenging activity of DPPH stable free radicals was measured as described previously [42]. In the case of the FEEs, a sample of 0.15 mL of the analyzed extracts was mixed with 2.85 mL of 0.3 mmol/l DPPH radical solution dissolved in 96% *v/v* ethanol. Measurement of absorbance at 517 nm against 70% (*v/v*) ethanol was performed after 10 min of incubation in the dark at room temperature using Hitachi UV-Vis Spectrophotometer U-5100. As a reference 6-hydroxy-2,5,7,8-tetramethylchroman-2-carboxylic acid (Trolox) was applied. The results are presented as Trolox equivalents (TEAC) in mmol Trolox/l.

The evaluation of ABTS radical scavenging activity was performed as described elsewhere [42]. The stock solution was a 7 mmol/L solution of ABTS (2,2’-azino-bis(3-ethylbenzothiazoline-6-sulfonic acid)) in a 2.45 mmol/L aqueous solution of potassium persulfate. After dissolving the components, the solution was incubated for 24 h, in the dark at room temperature, then diluted with 50% (*v/v*) methanol to obtain a working solution. An aliquot of 2.5 mL of working ABTS solution and 0.025 mL of an analyzed extract (FEEs) was introduced into the spectrophotometric cuvette. After 6 min incubation in dark at room temperature absorbance at 734 nm was measured. As previously, Trolox was used as a reference and the results were expressed as Trolox equivalents (TEAC) in mmol Trolox/l.

The assessment of total phenolic compounds content in extracts was performed spectrophotometrically using the Folin-Ciocalteu technique, according to the previously described method by Nowak et al. [42]. Shortly, to 0.15 mL of the test extract, 0.15 mL of tenfold diluted Folin-Ciocalteu reagent, 1.35 mL of 0.01 M sodium carbonate aqueous solution, and 1.35 mL water was added and mixed. The samples were incubated for 15 min at room temperature. The spectrophotometric measurement was carried out at 765 nm. Gallic acid (GA) was applied as a standard, and results were expressed as gallic acid equivalents (GAE) in mmol GA/l.

Moreover, measurements of the antioxidant activity and total polyphenol content were performed for BC-5%FEE, BC-10%FEE, and BC (control), samples were prepared according to the modified methods of Sukhtezari et al., Moradian et al. [17,18]. Shortly, 100 mg of each membrane was placed in the Eppendorf tube containing 2 mL of methanol and was stirred for 3 h at room temperature. Next, the supernatant collected was analyzed as above. For all methods, each extract was evaluated in triplicate.

The L929 murine fibroblasts, as model cells in biomaterials biocompatibility studies [76], were exposed to the extracts released from BC-5%FEE, BC-10%FEE, BC into the cell culture medium. The BC-5%FEE, BC-10%FEE as well as pure BC were punched into circular sheets of 6 mm diameter, sterilized by autoclaving at 126 °C for 11 min, and aseptically transferred to a 24-well plate. According to the previously described protocol by Subtaweesin et al. [77], the samples were soaked inappropriate cell culture medium (DMEM high glucose supplemented with L-glutamine, penicillin-streptomycin, and 10% heat-inactivated fetal bovine serum) and incubated at 37 °C for 24 h. The free medium was used as the control. Afterward, a medium containing the extracts (standardized for total polyphenol content) was collected and added to L929 murine fibroblasts, which had been seeded in a 96-well plate (5 × 10^3^ cells/well) the day before. Medium with the respective extract (without cells) was used as blank. After 24 h optical microscopy imaging of L929 cells was performed using Smart Fluorescent Cell Analyzer JuLi (Korea). Next WST-1 reagent was added, incubated for 30 min and absorbance was measured at 450 nm (with 620 nm background correction), using a spectrophotometric microplate reader (Infinite 200 Pro, Tecan, Switzerland). The cell viability was calculated using the following formula (1):

(1) $$The\;cell\;viability=\frac{\left(A_{test}-A_{blank}\right)}{\left(A_{control}-A_{blank}\right)}100\%$$

where:

*A_test_*—cells with medium containing the extracts,
*A_blank_*—medium with the respective extract (without cells),
*A_control_*—cells with a free medium.

The readings were acquired from three independent experiments.

The pigskin penetration of bioactive compounds through the skin from the BC-5%FEE, BC-10%FEE was evaluated using Franz diffusion cells (SES GmbH Analyse Systeme, Germany) with a diffusion area of 1 cm^2^. The acceptor fluid was phosphate buffer (pH = 7.4) kept at 37 °C with constant stirring using a magnetic stirrer. The volume of the receptor chamber used in the penetration tests was 8 mL. The solubility of the tested compounds was higher as compared to their concentration in the acceptor fluid. In the acceptor chamber constant temperature of 37.0 ± 0.5 °C via thermostat (VEB MLW Prüfgeräte-Werk type of 3280) was maintained. In the experiment, porcine skin was used because it has similar permeability to human skin [78]. The fresh abdominal porcine skin was washed in PBS buffer pH 7.4 several times. The skin thickness of 0.5 mm was excised and wrapped into aluminum foil and stored at −20 °C until use. Under these conditions, the skin was used for 3 months. This freezing time ensures the stability of the skin barrier properties [79]. Before the experiment, the skin was thawed at room temperature for about 30 min, and then it was soaked in a PBS solution for 15 min to hydrate it [80,81]. The undamaged skin pieces with an even thickness were used for experiments. The skin pieces were placed between the donor and acceptor chamber of Franz diffusion cells, then their integrity was checked. To measure skin impedance, a donor chamber with a capacity of 2 mL was installed. Skin impedance was measured using an LCR meter 4080 (Conrad Electronic, Germany), operated in parallel mode at an alternating frequency of 120 Hz (error at kΩ values < 0.5%). The tips of measuring probes were immersed in the donor and the second in acceptor chambers, both filled with PBS buffer pH 7.4 [82]. For the experiment, only the skin samples with impedance >3 kΩ were used. These values are similar to the electrical resistance for human skin [83]. After impedance measurement, the donor chamber was removed. The BC-5%FEE, BC-10%FEE as well as pure BC were cut to pieces that fitted the surface area (1 cm^2^) of diffusion. Next, BC was applied to the diffusion surface, then 100 µL PBS was applied on all BC. The penetration experiments were performed under occluded conditions by sealing the donor compartment with microscope coverslips [21,22]. 

The penetration experiment was carried for 24 h. The samples were collected after 0.5 h, 2 h, 3 h, 4 h, 5 h, 8 h, and 24 h. After this time samples of the acceptor fluid (0.6 mL) were withdrawn and acceptor chambers were refilled with fresh buffer at the same pH. The phenolic acid concentrations in the acceptor phase were measured by HPLC. After 24 h the BC-FEEs and BC and skin samples were removed from the Franz diffusion cell. The skin samples were carefully rinsed in PBS solution at 7.4 pH and dried at room temperature. In the next stage, skin samples were placed in 2 mL methanol and were incubated for 24 h at 4 °C. After this time skin samples were homogenized for 3 min using a homogenizer (IKA^®^T18 digital ULTRA TURRAX). The homogenate was centrifuged at 3500 rpm for 5 min. The supernatant was collected for subsequent HPLC analysis with pure methanol applied as a control. Accumulation of the phenolic acids in the skin was calculated by dividing the amount of the substances remaining in the skin by mass of skin sample and was expressed as the mass of phenolic acid per mass of the skin (µg/g skin). The cumulative mass of phenolic acids (μg cm^−2^) penetrated to acceptor fluid was calculated based on concentration determined by the HPLC method. The flux (in μg cm^−2^ h^−1^) of the phenolic acids into acceptor fluid was determined as the slope of the plot of cumulative mass in the acceptor fluid versus time. The release of studied compounds and their accumulation in pigskin was also assessed in pure BC (control). Results are presented as the mean ± standard deviation (SD). Statistical calculations were done using Statistica 13 PL software (StatSoft, Polska).

## 5. Conclusions

Bacterial cellulose membranes (BC) were used as a matrix for the entrapment of extract of *E. angustifolium*. The produced membranes were characterized by good structural and mechanical properties. Loading the membranes with this plant extract leads to an increase in antioxidant properties and the content of valuable ingredients such as phenolic acids. After the application of BC containing FEE membranes to pigskin a lot of bioactive compounds released from the membranes, were absorbed by the skin. Therefore, topical application of such membranes may be a promising tool for the local delivery of antioxidants to the skin.

## Figures and Tables

**Table 1:** Phenolic acid concentration, the total polyphenol content, and antioxidant activity of FEE. Mean value ± standard deviation, *n* = 3.

Evaluated Compound/Parameter	(mg/100 mL)
Chlorogenic acid (ChA)	26.78 ± 0.55
Gallic acid (GA)	78.02 ± 1.00
4- hydroxybenzoic acid (4-HA)	34.97 ± 0.07
3- hydroxybenzoic acid (3-HB)	12.64 ± 1.20
3,4-dihydroxybenzoic acid (3,4-DHA)	15.55 ± 0.38
Caffeic acid (CA)	7.13 ± 0.33
Total polyphenol content (mmol GA/l)	41.04 ± 0.10
DPPH (mmol Trolox/l)	19.36 ± 0.24
ABTS (mmol Trolox/l)	21.51 ± 0.86

**Table 2:** Results of mechanical tests for BC-FEE and BC. Mean values ± standard deviation, *n* = 5.

Sample	Young Modulus [MPa]	Elongation at Break [%]	Tensile Strength [MPa]
BC	13,807.88 ± 596.43	0.85 ± 0.34	115.53 ± 15.28
BC-5%FEE	20,974.64 ± 115.12	1.08 ± 0.16	137.38 ± 40.86
BC-10%FEE	11,327.83 ± 144.20	0.76 ± 0.14	76.48 ± 19.06

**Table 3:** Phenolic acid concentration, the total polyphenol content, and antioxidant activity of BC-5%FEE, BC-10%FEE, and BC. Mean ± standard deviation, *n* = 3.

		BC-5%FEE	BC-10%FEE	BC (Control)
Phenolic acid (µg/g membrane)	ChA	83.69 ± 2.57	140.52 ± 6.44	nd
	GA	275.44 ± 56.44	453.66 ± 6.95	nd
	4-HB	150. 31 ± 11.31	285.15 ± 21.28	nd
	3-HB	43.74 ± 4.83	72.50 ± 6.48	nd
	3,4-DHA	75.93 ± 1.13	116.17 ± 8.65	nd
	CA	31.83 ± 1.23	57.80 ± 4.12	nd
Total polyphenol content (mmol GA/l)		0.45 ± 0.01	0.63 ± 0.02	na
DPPH (mmol Trolox/l)		0.44 ± 0.05	0.55 ± 0.01	na
ABTS (mmol Trolox/l)		1.59 ± 0.01	2.09 ± 0.01	na

ChA—chlorogenic acid, GA—gallic acid, CA—caffeic acid, 4-HA—4-hydroxybenzoic acid, 3-HB—3-hydroxybenzoic acid, 3,4-DHA—3,4-dihydroxybenzoic acid; DPPH - 2-diphenyl-1-picrylhydrazyl; ABTS -2,2′-azino-bis(3-ethylbenzothiazoline-6-sulfonic acid), nd—no detected, na—no activity.

**Table 4:** The total phenolic content released from BC-5%FEE, BC-10%FEE, and BC to the culture medium and its effect on cell viability.

	BC + FEE5%	BC + FEE 10%	BC
Total polyphenol content (mmol GA/dm^3^)	0.29 ± 0.02	0.59 ± 0.09	nd
Cell viability (% of the control medium)	73.99 ± 7.14	14.16 ± 5.91	102.21 ± 3.73

nd—no detected.

**Table 5:** Phenolic acid concentration, the total polyphenol content, and antioxidant activity in acceptor fluid after 24 h penetration. Mean ± standard deviation, *n* = 6.

		BC + FEE5%	BC + FEE 10%	BC (Control)
Phenolic acid (µg)	ChA	1.28 ± 0.25	2.16 ± 0.430	nd
	GA	9.22 ± 0.84	12.26 ± 1.96	nd
	4-HB	5.07 ± 0.78	6.56 ± 0.09	nd
	3-HB	< 0.50	< 0.50	nd
	3,4-DHA	2.01 ± 0.23	3.56 ± 0.46	nd
	CA	< 0.50	1.40 ± 0.31	nd
Total polyphenol content (mmol GA/l)		0.016 ± 0.01	0.051 ± 0.01	na
DPPH (mmol Trolox/l)		na	na	na
ABTS (mmol Trolox/l)		0.084 ± 0.02	0.15 ± 0.03	na

ChA—chlorogenic acid, GA—gallic acid, CA—caffeic acid, 4-HA—4-hydroxybenzoic acid, 3-HB—3-hydroxybenzoic acid, 3,4-DHA—3,4-dihydroxybenzoic acid; DPPH—2-diphenyl-1-picrylhydrazyl; ABTS -2,2′-azino-bis(3-ethylbenzothiazoline-6-sulfonic acid), nd—no detected, na—no activity.

**Table 6:** The parameters characterizing phenolic acids transport through the pigskin after application of BC-FEEs in penetration study.

Phenolic Acid	BC-5%FEE			BC-10%FEE		
	J_SS_, μg cm^−2^ h^−1^	K_P_ 10^−5^, cm h^−1^	L_T,_ h	J_SS_, μg cm^−2^ h^−1^	K_P_ 10^−5^, cm h^−1^	L_T,_ h
ChA	-^iv^	-^iv^	~5	0.267 ± 0.021	379.315 ± 36.493	2.379
GA	0.389 ± 0.096	309.168 ± 76.150	2.009	0.549 ± 0.079	242.032 ± 34.83	1.479
4-HB	0.389 ± 0.043	517.582 ± 57.202	2.444	0.345 ± 0.008	241.976 ± 5.516	1.359
3-HB	-^iv^	-^iv^	~24	-^iv^	-^iv^	~5
3,4-DHB	0.195 ± 0.031	512.116 ± 81.140	1.820	0.345 ± 0.019	593.944 ± 33.447	1.359
CA	-^iv^	-^iv^	2.204	0.138 ± 0.022	477.516 ± 77.129	1.377

^iv^—immeasurable value.

**Table 7:** Amount of the phenolic acids, the total polyphenol content, and antioxidant activity of fluid after skin extraction collected after 24 h penetration, *n* = 6.

		BC + FEE5%	BC + FEE 10%	BC (Control)
Phenolic acid (µg/g skin)	ChA	30.77 ± 0.95	41.05 ± 1.99	nd
	GA	151.34 ± 13.85	222.94 ± 16.60	nd
	4-HB	45.36 ± 1.33	127.67 ± 1.59	nd
	3-HB	17.93 ± 0.93	30.62 ± 4.06	nd
	3,4-DHA	31.91 ± 0.62	52.03 ± 5.11	nd
	CA	21.58 ±1.42	41.18 ± 3.16	nd
Total polyphenol content (mmol GA/l)		0.33 ± 0.01	0.44 ± 0.01	na
DPPH (mmol Trolox/l)		0.27 ± 0.005	0.39 ± 0.01	na
ABTS (mmol Trolox/l)		1.02 ± 0.01	1.52 ± 0.06	na

ChA—chlorogenic acid, GA—gallic acid, CA—caffeic acid, 4-HA—4-hydroxybenzoic acid, 3-HB—3-hydroxybenzoic acid, 3,4-DHA—3,4-dihydroxybenzoic acid; DPPH—2-diphenyl-1-picrylhydrazyl; ABTS -2,2′-azino-bis(3-ethylbenzothiazoline-6-sulfonic acid), nd—no detected, na—no activity.

**Table 8:** The FEE content in each BC-membranes applied in the study.

Sample	mg FEE/g Membrane *
BC	-
BC-5%FEE	465.0
BC-10%FEE	857.8

* The pure FEE content in the membrane, calculated after drying the membranes at 40 °C in a ventilated oven for 12 h.

## Data Availability

The data presented in this study are available on request from the corresponding author.
